# An unsupervised image segmentation algorithm for coronary angiography

**DOI:** 10.1186/s13040-022-00313-x

**Published:** 2022-10-21

**Authors:** Zong-Xian Yin, Hong-Ming Xu

**Affiliations:** Department of Computer Science and Information Engineering, Southern Taiwan University of Science and Technology, Tainan, Taiwan

**Keywords:** Classification, Classifier, Bio and medical imaging, Image segmentation, Machine learning, Pattern recognition

## Abstract

Computer visual systems can rapidly obtain a large amount of data and automatically process them with ease. These characteristics constitute advantages for the application of such systems in the automatic analysis of medical images, as well as in processing technology. The precision of image segmentation, which plays a critical role in computer visual systems, directly affects the quality of processing results. Coronary angiographs feature various background colors, complex patterns, and blurry edges. The image areas containing blood vessels cannot be precisely segmented through regular methods. Therefore, this study proposed an unsupervised learning algorithm that uses regional parameter expansion (RPE). This method was derived from the flood fill algorithm, which can effectively segment image areas containing blood vessels despite a complex background or uneven light and shadow. An optimal cover tree (OCT) algorithm was proposed for the establishment of coronary arteries and the estimation of vessel diameter. Through the region growing method, spanning trees were used to record the cover length of adjacent connections, thereby establishing vessel paths, and the length can be used to track changes in vessel diameter.

## Introduction

According to World Health Statistics Overview 2019, coronary artery disease [1] ranks among the top 10 causes of mortality worldwide and constitutes a major reason for hospitalization. The main coronary artery consists of three branches, namely, the right coronary artery, left circumflex artery, and left anterior descending (LAD) artery, which supply blood to the entire body. Narrowing or blocking any one of these arteries may reduce the supply of nutrients and oxygen, preventing normal cardiac output and inducing complications such as heart failure or arrhythmia. Coronary artery disease has multiple manifestations that vary depending on the mechanisms of pathogenesis.

Several clinical examinations are used to detect coronary artery disease. For example, exercise electrocardiography is used to test for oxygen deficiency in the cardiac muscles [2], computed tomography can be used to detect coronary artery calcification [3], and coronary angiography can identify the occurrence location and severity of coronary artery stenosis [4]. Coronary angiography is considered the safest and most advanced of the various invasive techniques. Specifically, a contrast agent is injected through catheterization, and then X-ray imaging and recording are conducted to assess heart function and examine vascular conditions, including for the identification of any vascular lesions. Physicians can make precise diagnoses of heart conditions through this method. According to the severity of coronary artery narrowing, suitable treatments or interventions are recommended.

In medicine, using computers in medical image processing for fast, accurate analysis and processing has become a development trend in the diagnostic assistance context. Medical image identification and analysis have unique advantages; they promote physician–patient communication on clinical information and provide accurate medical information within a short period of time. Because coronary angiographs often contain complex patterns and blurry edges, their segmentation cannot be optimized through a single method; typically, one method can only resolve one specific image problem. Numerous researchers have proposed various solutions, the practical application of which has not realized ideal results. To assist physicians in decision-making for clinical diagnosis, a system based on regional parameter expansion and an optimal cover tree algorithm was developed in this study. The system can be used to calculate the vessel diameter changes in coronary angiographs. A regional parameter expansion algorithm was effectively applied in segmenting the image areas containing major blood vessels. The optimal cover tree algorithm developed successfully tracked vessel diameter in the segmented images. Different diameters are indicated with different colors to indicate the vessel diameter changes in various areas.

### Literature review

Neural networks have been applied in medical image processing. For example, in [5–7], the researchers integrated neural networks with machine learning techniques and applied them to the classification, prediction, and diagnosis of breast cancer. A comparison with various algorithms revealed the high accuracy of the neural networks. Zhang [8] integrated GoogLeNet with transfer learning to pretrain 1.2 million images, experimentally applying the model to the automatic classification of four skin diseases. The accuracy exceeded 85.86%. Wang et al. [9] developed an image classifier for dividing all images in an electronic health record system into a lexicon of the following subclasses: lesions, nonlesional partial body photographs, dermoscopy, surgical images, labels and forms. Four different network architectures, InceptionV3, Xception, InceptionResNetV2, and VGG16, were compared. The overall accuracy reached 75%. Wu et al. [10] analyzed five common neural network structures, conducting clinical image recognition for six common facial skin diseases. The CNN networks tested effectively identified the conditions.

Shi et al. [11] trained CNNs in the learning of chest computed tomography scans of patients with tuberculosis. The framework consists of two stages. The first, the region proposal generation stage, laid anchors of varying scales on the feature map after convolutional layers of VGG16 were used for prediction, taking into account variations in pulmonary nodule diameter (from approximately 3 to 30 mm). The second stage, false positive reduction, involves removing the large number of false positive nodules from the first phase and generating the object categories, as well as determining the size of the location of objects. Kang et al. [12] trained a CNN model to examine positron emission tomography images of the brains of patients with Alzheimer’s disease. The images were classified visually according to the brain amyloid plaque load score. The VGG16 model was used to examine the gray matter, with gray matter masking (GMM) applied to the standard slice-based samples. All the performance metrics were higher with GMM than without GMM. Calderon et al. [13] compared eight CNN structures and performed automatic quality assessment of retinal images taken by a nonmydriatic fundus camera. The VGG16 model was determined to have the optimal performance, as indicated by its 100% precision. Thao et al. [14] used CNNs to analyze skin tumors, employing four convolutional blocks and one fully connected classifier and integrating transfer learning into the VGG16 model. Pretrained parameters were used to reduce the calculation complexity in VGG16. The tumors in the images were effectively classified as melanocytic and nonmelanocytic.

An image usually only has certain regions, called the foreground or the target, that are meaningful or interesting for their capture of people or objects. The remainder of the image is called the background. The vessel-containing sections of coronary angiographs that are meaningful for analysis and recognition are called the foreground, and the rest, represented by different shades of colors, are called the background. Image segmentation precision affects subsequent image processing and analysis and is critical for clinical diagnosis. The main image segmentation methods are classified as region-based, edge-based, and segment-based. This paper discusses studies on image segmentation methods involving unsupervised classification and region growing–based image segmentation methods, among which the k-means algorithm is the most commonly used. Mohd et al. [15] applied k-means clustering to thermal infrared images to segregate and separate the hot spot probability layer from the background layer. The image was then converted from an RGB color space into the L*a*b* color space, and every pixel was labeled using the k-means algorithm. Kulshreshtha et al. [16] proposed steps of feature extraction using local binary patterns and k-means clustering. Mammograms were cropped depending upon their region of interest (ROI). The k-means algorithm generated the clusters on the basis of the visual similarity of the ROIs. Query image features were matched with all cluster representatives to find the closest cluster, from which abnormal patterns were then retrieved. Yadav et al. [17] used the k-means algorithm to segment medical images by color. Specifically, brain images were segmented into *n* groups. Finally, a support vector machine classifier was used to classify the tumor-containing images, with favorable results. Obaid et al. [18] integrated the Chan–Vese and k-means algorithms to segment tomography images of the liver. The normal tissues around the liver have intensity values comparable to those of the liver; this can lead to oversegmentation. The Chan–Vese algorithm first isolated the image of healthy tissues around the lesion. The k-means algorithm was then used to segment the location of the lesion.

For the accurate segmentation of the coronary arteries in angiography, Kulathilake et al. [19, 20] proposed computing the difference between two consecutive frames of the coronary cineangiogram input to generate a foreground mask. Morphological erosion and flood filling were then used to isolate and extract the vessel regions from the mask. AbdelRaouf et al. [21] investigated the identification of narrowed coronary arteries from angiographic scans. They integrated the Frangi filter with the region growing algorithm for coronary artery segmentation. Next, the k-nearest neighbors algorithm was used to separate lumen diameter into four blockage stages. Wang et al. [22] applied a Hessian filter to clarify vascular structure in images. Subsequently, k-means and region growing algorithms were used for the segmentation of vessel-containing regions from the images. Huang et al. [23] used a statistics-based threshold segmentation algorithm to compute image threshold values, which divide images into regions. A three-dimensional region growing algorithm was applied to the segmentation of three-dimensional abdominal images of liver segmentation. To avoid a wrong threshold that could have disastrous results in image segmentation, the multi-level thresholds technique was used to separate grayscale pixels into several regions corresponding to one background and multiple objects. Singh et al. [24] proposed the Learning enthusiasm-based teaching–learning-based optimization (LebTLBO). The LebTLBO simulated the behaviors of the teaching and learning process in a classroom, and introduced two modules: the learning enthusiasm-based teacher part and the learner phase. The approach gives the probability of getting the amount of information by the learner from the teacher. Singh et al. [25] also proposed another hybrid version of the Dragonfly algorithm (DA) and Firefly Algorithm (FA) approach (HDAFA). The approach simulated the behaviors of dragonfly’s swarming behavior and fireflies’ social behavior. The approach has the search capability with the ability to exploit to obtain an ideal global solution. Experimental outcomes demonstrate both of the approaches are highly efficient in fast convergence and image segmentation quality.

## Methods

**Fig. 1:**
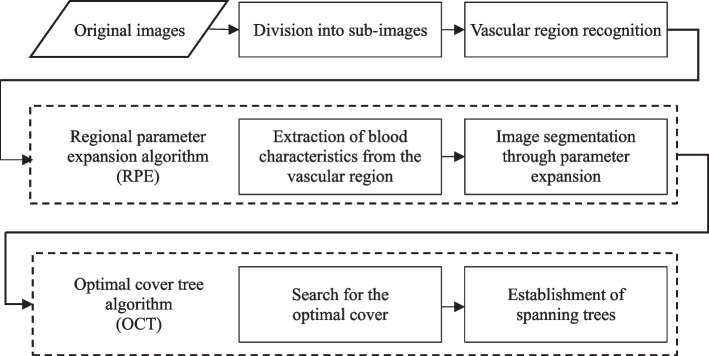
Flow chart of our proposed method

In this paper, we introduce a method for calculating changes in vessel diameter in coronary angiographs by using regional parameter expansion and an optimal cover tree algorithm. In this method, regional characteristics are first obtained to segment the vascular regions in the images. Next, the optimal cover tree algorithm is used to establish the sequential relationships between the vascular regions and to measure the changes in the diameters of the blood vessels. Figure 1 presents a flow chart of the method, which consists of five steps. In step 1, images are divided into subimages of fixed size for image recognition. In step 2, deep learning is used for vascular region recognition. In step 3, a regional parameter expansion (RPE) algorithm is used to segment the vascular regions in the subimages. In step 4, an optimal cover tree (OCT) algorithm is used to examine and connect the vascular regions in the adjacent subimages. In the final step, visualization processing is performed according to changes in vessel diameter.

### Image division into subimages

The original images were 512 × 512 coronary angiographs obtained from hospitals. Some of the original images were first segmented into 32 × 32 subimages, where *p*_*i*, *j*_ *i* = 1, ⋯16, *j* = 1⋯16. Subimages containing blood vessels were selected as the positive samples for training data. The remaining subimages were negative samples. Ultimately, a total of 981 positive samples and 5163 negative samples were obtained. Furthermore, segmented images were divided into 16 × 16 or 64 × 64 subimages. The oversegmentation of the blood vessels in the 16 × 16 subimages complicated the selection of the positive samples. The 64 × 64 subimages contained not only blood vessels but also an excessive amount of background noise. Therefore, we selected the 32 × 32 subimages, which best facilitated the segmentation of blood vessels with the background, for further analysis.

### Vascular region recognition

This step involved training the deep learning neural network for the recognition of vascular subimages. VGG16 outperformed VGG19, ResNet, and GoogLeNet in prediction; thus, it was selected for vascular subimage classification. After model training, VGG16 was used to recognize the testing samples, and the subimages were classified as *DL*_*A*_ or ${DL}_{\overline{A}}$ (vascular and nonvascular, respectively).

### Image segmentation through the regional parameter expansion algorithm (RPE algorithm)

The accuracy rate for *DL*_*A*_, recognized using deep learning, can exceed 95%. Therefore, the segmentation of blood vessels from the background of the images was performed using the k-means algorithm, where *k* = 2, to calculate the optimal characteristic parameter. This is represented by the following equation: δ_*i*, *j*_ *i* = 1⋯16, *j* = 1⋯16. Because the grayscale values of vascular regions are smaller in coronary artery imaging, pixel groups with small grayscale values after grouping are considered vascular regions. However, some vascular subimages can be misclassified as ${DL}_{\overline{A}}$ because of an overly small vascular region ratio or the interference of the background noise. These overlooked vascular areas can affect the precision of blood vessel diameter calculation.

We used the regional parameter expansion algorithm, which can prevent factors such as complex backgrounds or uneven light and shadow from hindering the segmentation of vascular subimages. This method is an extension of the flood fill algorithm. Regarding the algorithm procedure, the subimage of *ID*_*A*_ was first regarded as the center of the region to identify a characteristic that could be used for vascular image identification. The characteristic was then expanded to the eight adjacent subimages for vascular image segmentation, thereby obtaining the total vascular area in this 3 × 3 grid. This method corrected the misidentification error in the deep learning step, thereby ensuring the retention of the vascular images. Table 1 lists the pseudocode of the RPE algorithm. In the first step, a subimage *p*_*i*, *j*_ was taken from the *ID*_*A*_ set. The k-means algorithm was used to obtain the characteristic parameter δ_*ij*_ of the vascular pixel set and the background image set. Next, this characteristic parameter was used in the eight connected subimages for image segmentation; thus, the vascular images in this 3 × 3 grid were obtained. This procedure was repeated until all subimages in *ID*_*A*_ had been examined.

**Table 1:** Regional parameter expansion algorithm

FOR each *p* in *ID*_*A*_ Use the k-means algorithm (*k* = 2) to calculate the optimal threshold *δ*_*p*_. Apply *δ*_*p*_ to the eight adjacent sub-images: *p*_*i* − 1, *j* − 1_, *p*_*i*, *j* − 1_, *p*_*i* + 1, *j* − 1_, *p*_*i* − 1, *j*_, *p*_*i* + 1, *j*_, *p*_*i* − 1, *j* + 1_, *p*_*i*, *j* + 1_, *p*_*i* + 1, *j* + 1_, where i = 1⋯16, j = 1⋯16, for vascular image segmentation END FOR	

### Establishment of the optimal cover tree (OCT algorithm)

The OCT algorithm is a new algorithm proposed in this paper. It consists two steps of searching for optimal cover and establishment of spanning tree. The optimal cover searching step is a modified version of fuzzy cover-based clustering algorithm by Chiang [26]. The algorithm is to find minimal number of fuzzy covers to enclose the samples. Our OCT algorithm is to find the longest optimal cover tree, where the tree can be used to locate coronary arteries and remove noise.

**Fig. 2:**
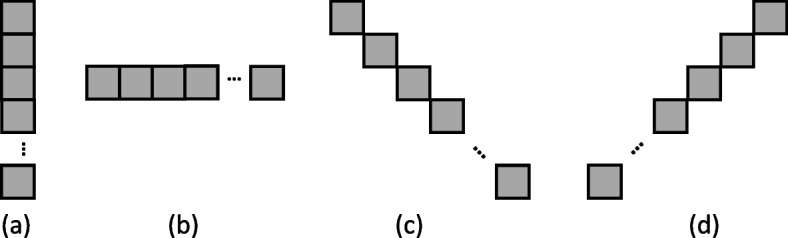
Five cover directions in the OCT algorithm, (**a**) vertically down and vertically up, (**b**) horizontal, (**c**) 45°to the right, and (**d**) 45° degrees to the left

In the paper, a cover is defined as a shortest distance between two black pixels along the edge of the black region in the binarized images. The OCT algorithm was used to scan the entire image for black regions in the following order: top–down and left–right from the top left pixel of the image. When black pixels were encountered, they were used as one end of the cover to calculate the optimal cover. To simplify the calculation, we used five cover directions, namely, vertically down, vertically up, horizontal, 45° to the right, and 45° to the left (Fig. 2). After calculating the optimal cover, the algorithm followed the black region downward or rightward to find the next black pixel along the edge and to calculate the next optimal cover. This process was continued until no more connected black regions remained. The algorithm then returned to scan the entire image to find any black regions that had not been covered. This process was repeated until all black regions were covered. Through calculation, the optimal cover was used to estimate the diameter of each vascular segment.

**Fig. 3:**
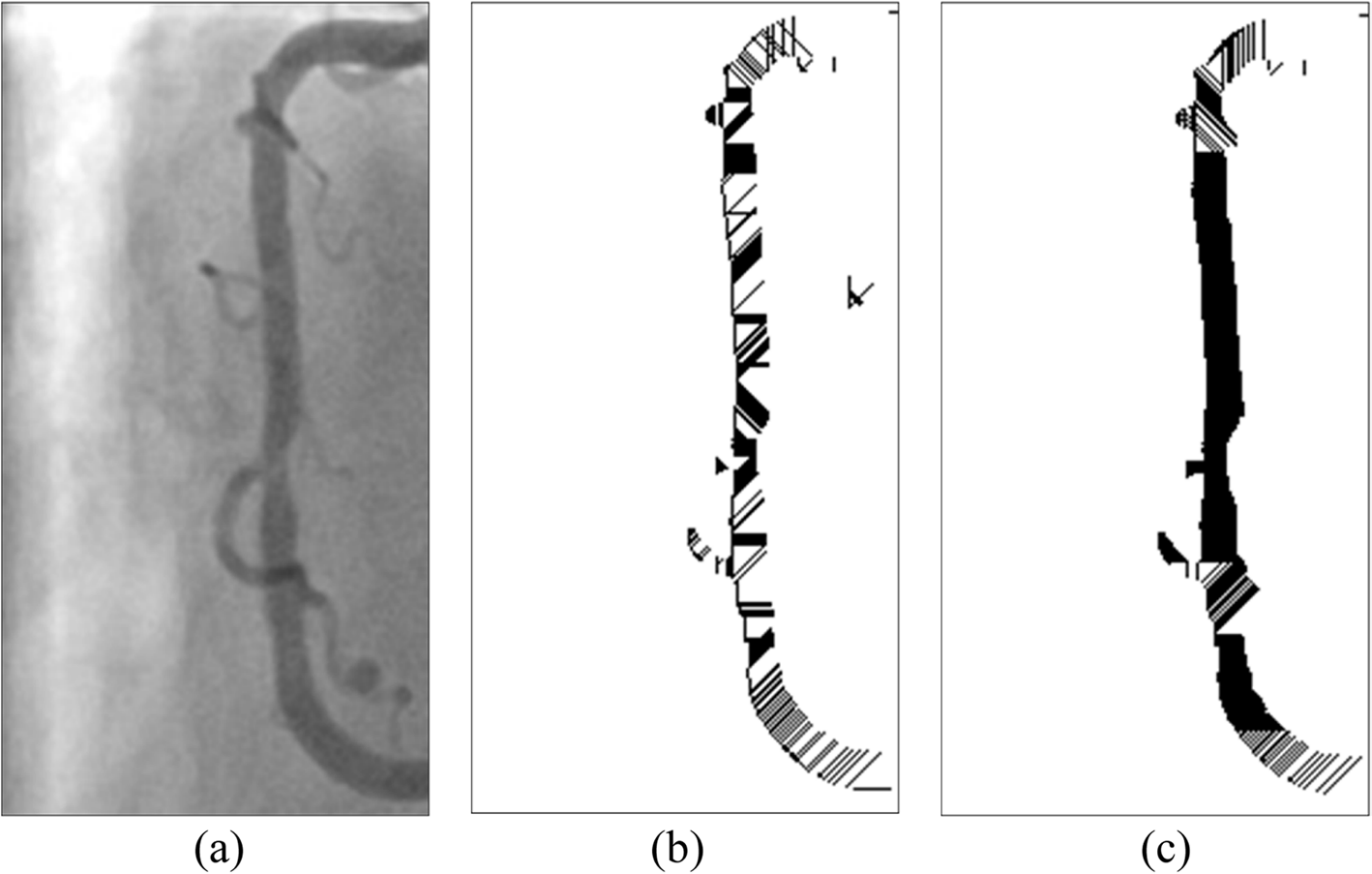
The edges of the blood vessels were usually uneven and jagged when the images underwent binarization. (**a**) the example blood vessel clearly extends vertically in the coronary angiograph, (**b**) only the shortest cover from the edge of each pixel was considered. The cover direction was complex thus affecting the accuracy with which the vessel diameter changes were traced, (**c**) presents the application results of the cover selection conditions. The middle section appears black since it was covered by a continuous, dense horizontal cover

However, when the images underwent binarization, the edges of the blood vessels tended to become uneven and jagged. This noise resulted in errors in optimal cover length selection. In Fig. 3(a), which represents a coronary angiograph, the main blood vessel clearly extends vertically. Figure 3(b) and (c) are the results of optimal cover implementation after binarization. Regarding Fig. 3(b), because only the shortest cover from the edge of each pixel was considered, its cover direction was complex, complicating the determination of the actual vascular direction and thus affecting the accuracy with which the vessel diameter changes were traced. Therefore, two selection conditions for cover were added as follows.

Rule 1: The angle between two adjacent cover directions may not exceed 45. (1).

Rule 2: The length difference of two adjacent covers must be within the standard error. (2).

To determine the optimal cover, the cover was compared with the previous adjacent cover. The angle of the two must not exceed 45°. Moreover, the length was decided according to whether it was within the differences between the adjacent cover length and the threshold. When these two conditions were not both met or when multiple covers satisfied them, the cover of the same angle as the adjacent row was prioritized for use. Otherwise, the cover direction of the shortest distance in that row was selected. Figure 3(c) presents the application results of the cover selection conditions. The middle section was covered by a continuous, dense horizontal cover; thus, an entire vascular segment appeared black.

**Fig. 4:**
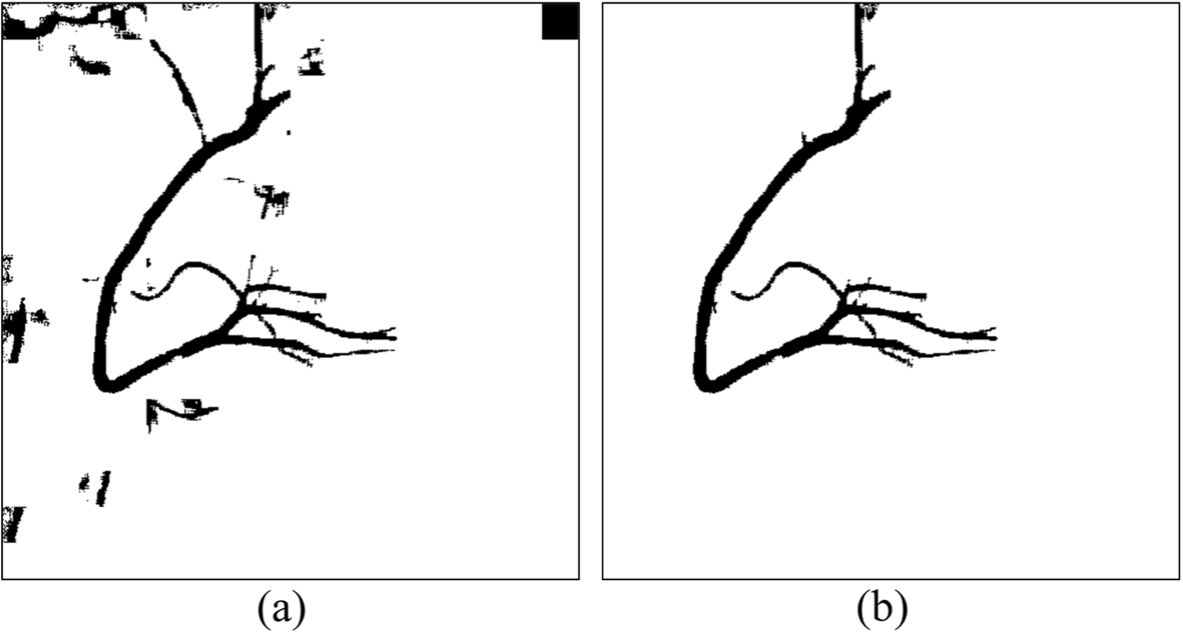
An example in which the OCT algorithm was implemented to establish a complete spanning tree and then eliminate the unconnected ones. (**a**) the original binary image, (**b**) the result, only the complete spanning tree with the greatest height was retained

**Fig. 5:**
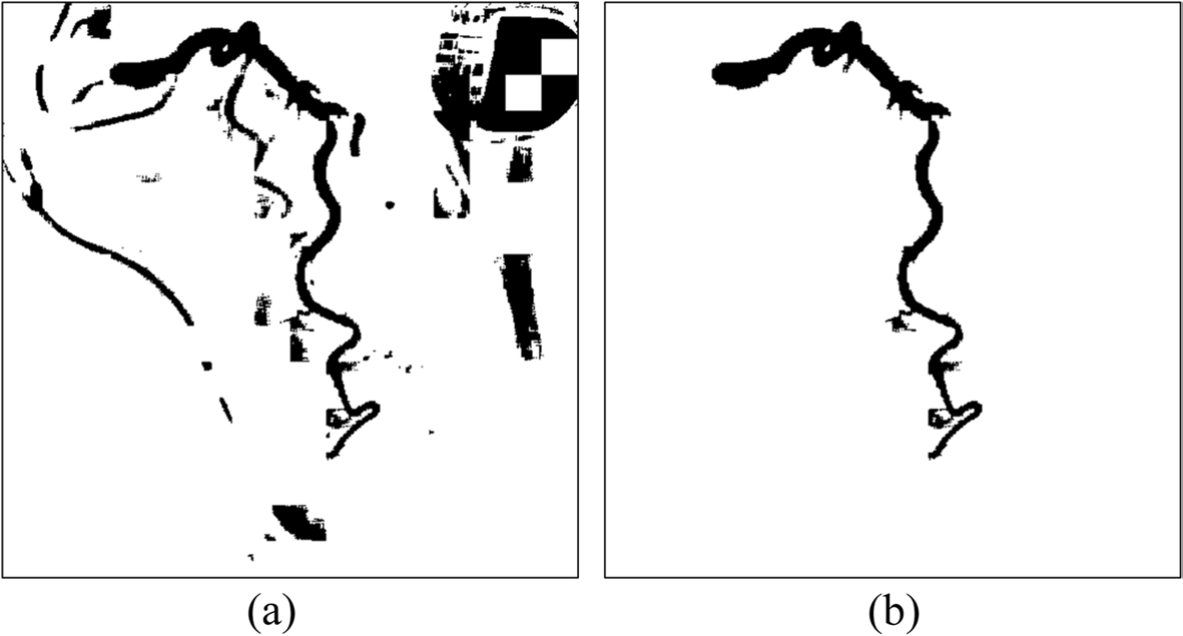
Images present assistive devices can be removed. (**a**) the original binary image, (**b**) the result, a blood vessel on top left was also removed because it did not connect to the main blood vessel

However, not all the covered areas were vascular. After image binarization, many black areas that contain noise must be deleted. As mentioned, the OCT algorithm, which used the region growing method, was applied [27]. The spanning tree method was used to record the cover of adjacent connections. Specifically, the nodes recorded the cover angle, cover length, and location, among other information. On the basis of the cover information stored in the nodes, the cover could be recreated to construct a complete vascular path. If two spanning trees had nodes that were adjacent covers, this meant that the two regions were connected and could be merged. By contrast, if a spanning tree could no longer be merged with other spanning trees, that meant it was complete. After the trees in the forest gradually merged into complete spanning trees, only the complete spanning tree with the greatest height was retained. The trees that did not connect with these trees were regarded as noise and removed from the binary image. Figure 4 presents a case in which the cover tree algorithm was implemented to establish a complete spanning tree and then eliminate the unconnected ones. Figure 4(a) is the original binary image. Figure 4(b) is the result of the implementation of the OCT algorithm. Most unconnected black regions were removed. However, a blood vessel on top left was also removed because it did not connect to the main blood vessel. Regarding the results of the implementation of the optimal cover tree algorithm in the binary image (a), as shown in Fig. 5(b), even images presenting assistive devices were removed

Table 2 presents the optimal cover tree algorithm. The steps are as follows: A pixel at the edge of a black region was identified, and the optimal cover was found. A spanning tree *ST*_*p*_ was established, and the cover was added. The search for the optimal cover for the eight adjacent subimages continued, and the cover was added to the *ST*_*p*_. After the spanning tree was complete, its nodes were examined to determine whether they were connected to another spanning tree. If so, the two spanning trees were merged. After the spanning trees in all the black regions had been constructed, the spanning tree with the greatest height was retained, and the others were deleted.

**Table 2:** Optimal cover tree algorithm

FOR each pixel *p* in *Graph*_*bin*_ IF (*p* is black in a region *BR*_*p*_) AND NOT(4 neighbors of *p* are black) search for the best cover CV_*p*_ of p generate a spanning tree ST_*p*_ and add CV_*p*_ to it FOR each NOT covered pixel *pb* in *BR*_*p*_ IF (*pb* is black) AND NOT(4 neighbors of *pb* are black) search for the best cover CV_*pb*_ of *pb* add CV_*pb*_ to ST_*p*_ END IF END FOR END IF FOR each tree *cv* in the spanning tree set CV IF *cv* has nodes adjacent to nodes in ST_*p*_ add ST_*p*_ to *cv* END IF END FOR END FOR search for the tree ST_*blood*_ which has the longest path	

## Results

### Vascular image identification through direct image segmentation involving clustering algorithms

**Fig. 6:**
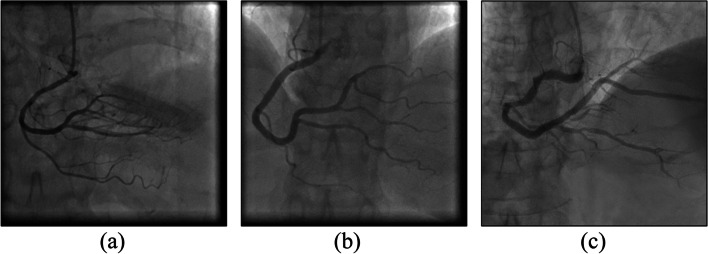
Original images

**Fig. 7:**
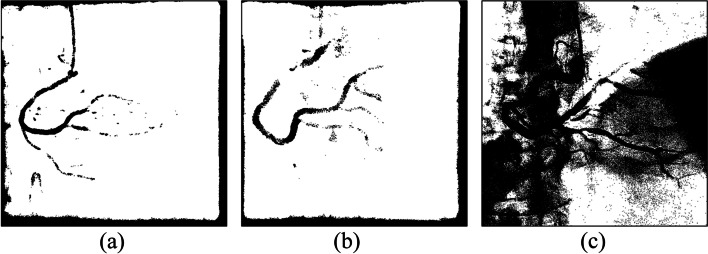
Results of image segmentation with the k-means clustering algorithm. (**a**) Vascular paths clearly revealed without severe fractures, (**b**) the vessels were fractured in multiple places or covered by background noise, and (**c**) the vessels could not be differentiated

**Fig. 8:**
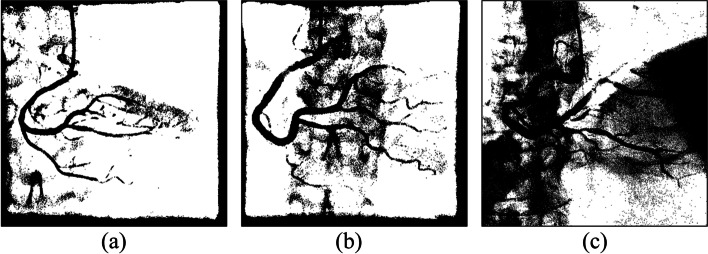
Results of image segmentation with the fuzzy c-means clustering algorithm. (**a**) Vascular paths clearly revealed without severe fractures, (**b**) the vessels were fractured in multiple places or covered by background noise, and (**c**) the vessels could not be differentiated

**Fig. 9:**
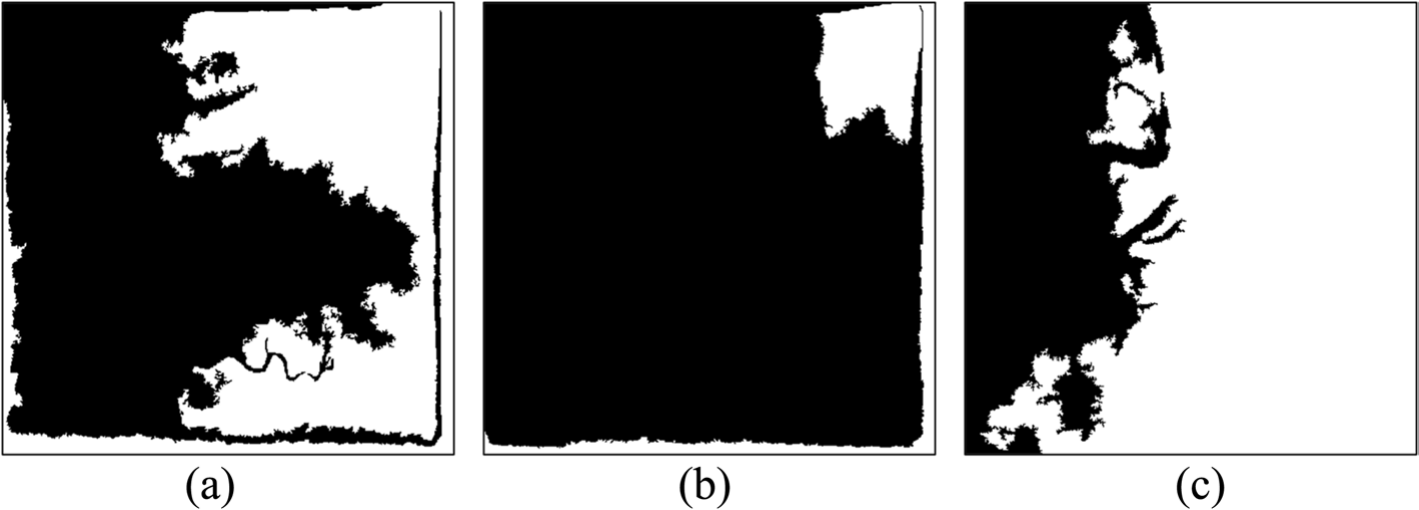
Results of image segmentation with a hierarchical agglomerative clustering algorithm. The vascular paths in (**a**), (**b**), and (**c**) cannot be differentiated

We experimented with various clustering algorithms to evaluate segmentation performance on the coronary angiographs. The experiment involved three algorithms, specifically the k-means clustering and fuzzy c-means (FCM) clustering [28] algorithms, as well as a hierarchical agglomerative clustering (HAC) algorithm [29]. To compare their performance with regard to regional characteristic–based image segmentation, we first applied these algorithms to the original coronary artery images. The experiment involved the segmentation of 173 different original images. Figure 6 presents three original images, and Figs. 7, 8 and 9 present the results of the respective application of the k-means, FCM, and HAC algorithms on those images.

The segmentation results of the original image were divided into three groups, namely, A, B, and C. The performance of the three algorithms was subjected to cross-analysis (Table 3). Images in group A clearly revealed vascular paths without severe fractures (Figs. 7(a) and 8(a)). For the group B images, as shown in Figs. 7(b) and 8(b), although the vascular paths were identifiable, the blood vessels were fractured in multiple places or covered by background noise. In this group, difficulties were encountered in vessel diameter calculation. Group C images contained the greatest number of images. After image segmentation, the vessels could not be differentiated, and subsequent treatments failed to improve these outcomes. As shown in Table 3, directly segmenting the original image was not feasible. Although the group A images were clear, they only accounted for 1% of the total. Group B accounted for 8% of the images. Although background noise was present, subsequent processing allowed for vessel diameter estimation. Overall, most of the images belonged to group C - presenting fractured blood vessels or complete coverage by black regions that were not amenable to subsequent processing. In short, vascular image areas could not be correctly identified through direct image segmentation involving clustering algorithms.

**Table 3:** Group comparison of the images’ strengths and weaknesses by algorithms

	Group A	Group B	Group C
k-means	2	15	156
FCM	3	18	152
HAC	0	0	173

### Subimage recognition with different deep learning models

The present algorithm first segmented the original images into subimages. Deep learning methods were then used to identify whether the subimages contained blood vessels. As mentioned, the performance of VGG16, VGG19, ResNet, and GoogLeNet was compared (Table 4), and VGG16 proved superior. Although it had a low recall rate, the use of the optimal cover tree algorithm allowed the merging of the misidentified vascular subimages, thereby increasing the accuracy of vessel diameter calculation.

**Table 4:** Model performance by indicator

	Accuracy	Precision	Recall	F1-Measure
VGG16	95%	90%	77%	83%
VGG19	92%	70%	84%	76%
ResNet50	94%	78%	82%	80%
GoogLeNet	86%	58%	61%	59%

### Regional parameter expansion strategies in subimage segmentation

**Fig. 10:**
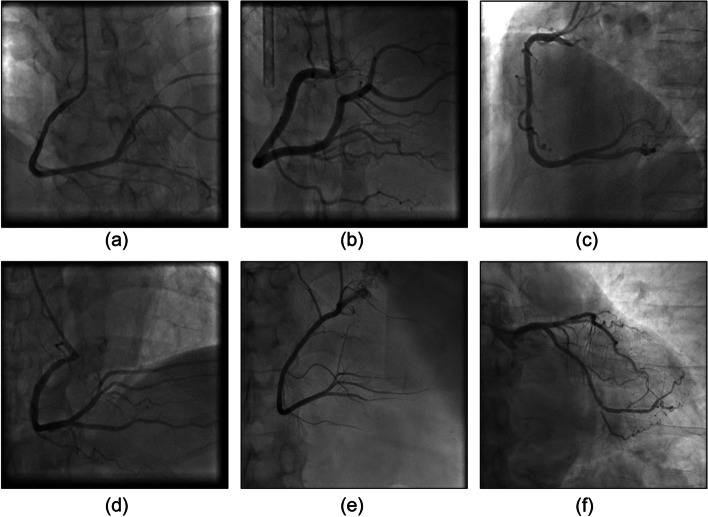
Original images

**Fig. 11:**
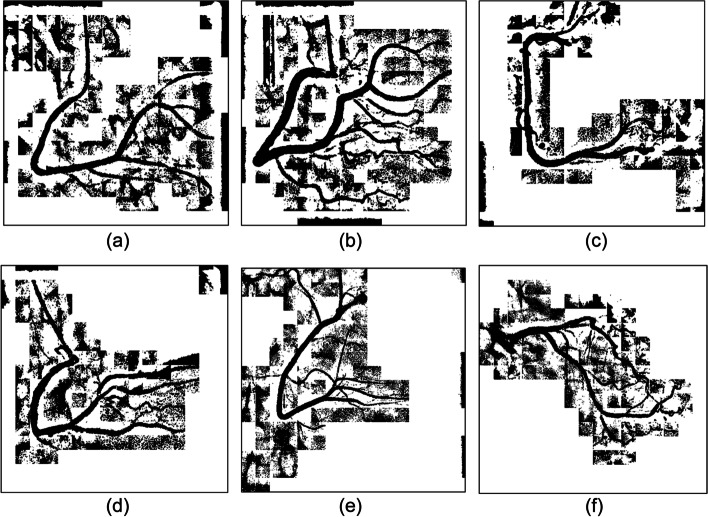
Results of single subimage segmentation

**Fig. 12:**
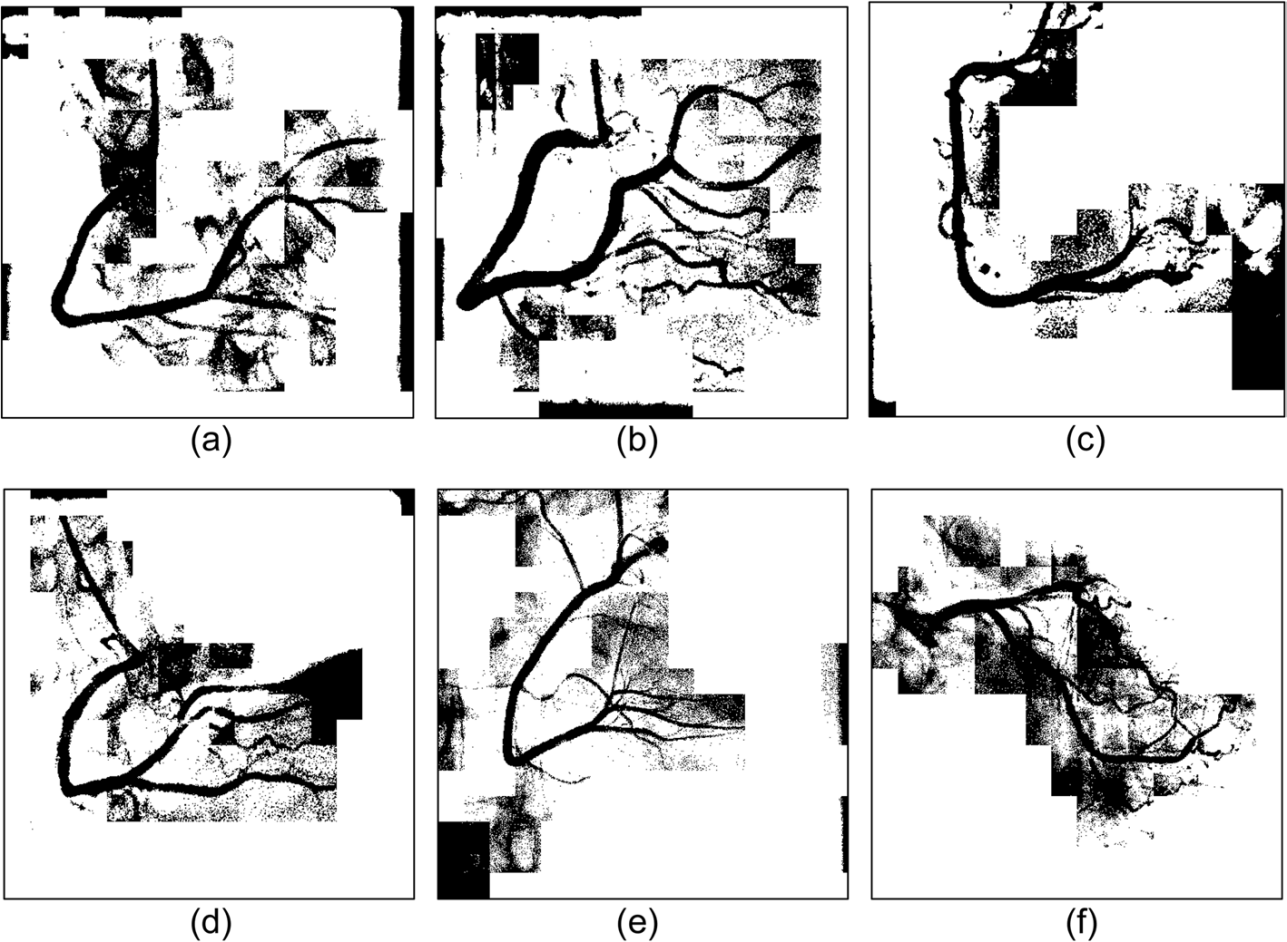
Integration of the central subimage and the eight adjacent subimages before image segmentation

**Fig. 13:**
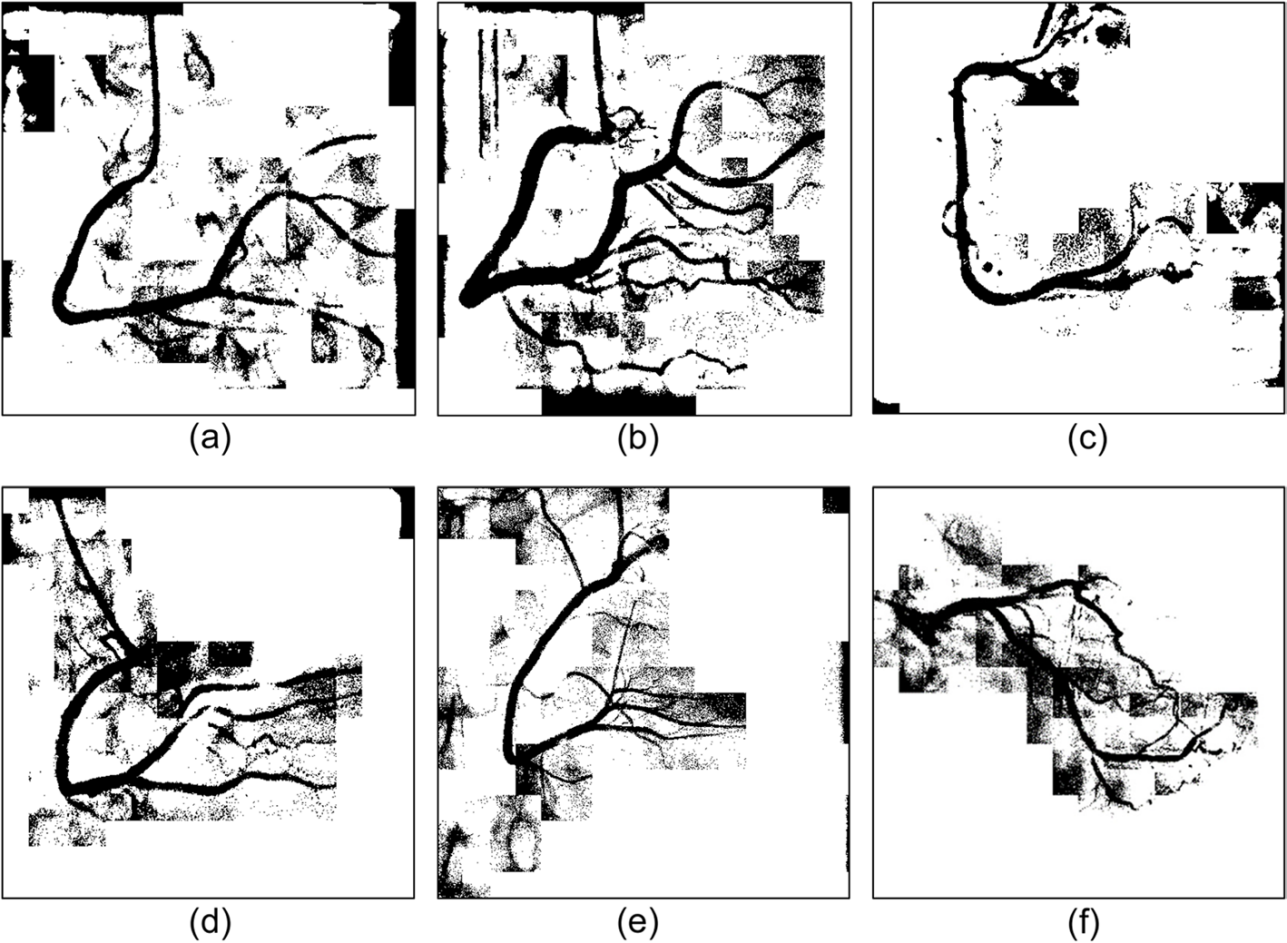
Results of segmentation with the eight adjacent subimages by using the flood fill algorithm

We experimented with three regional parameter expansion strategies. Central vascular subimages and their eight adjacent subimages were segmented, and the results were presented as an original-size image for comparison. The strategies are described as follows:

1. Segmentation of each subimage
  In this experiment, the k-means clustering algorithm was used to segment each subimage. Figure 10 shows six experimental cases. As shown in Fig. 11, vessel-containing areas were effectively segmented, but a considerable amount of background noise was generated in the process. This noise, observable in parts (b), (e), and (f) of the figure, complicated vessel identification. The main reasons were that the vascular region accounted for a small portion of the total area or that the grayscale values were overly similar to those of the background. These situations occurred frequently at the edge of blood vessels in the subimages. Using the k-means clustering algorithm forced the subimage pixels into two types of clusters, resulting in an excessive amount of noise and increasing the difficulty of noise processing and diameter calculation.
2. Merge of the central subimage with its eight adjacent subimages before image segmentation
  Under this strategy, central vascular subimages were first merged with their eight adjacent subimages into one image. Next, k-means image segmentation was performed. In the extraction of the characteristic values, this method was more susceptible to interference by the background of the original image than was the first strategy. As shown in Fig. 12(a), (d), and (f), for example, distinguishing vascular regions from the segmentation results was challenging. Overall, this strategy was associated with substantially less noise than the first strategy but had the disadvantage of being easily affected by the background.
3. Segmentation of adjacent subimages through the flood fill algorithm
  Vascular subimages were segmented with the k-means clustering algorithm used to calculate the threshold value for differentiation between the vessels and the background. Using this threshold value, the flood fill algorithm was then applied to the eight adjacent subimages, with the vascular subimage as the center. As shown in Fig. 13(a), (b) and (c), the vessels were segmented clearly and contained little noise. This method integrated the advantages of the previous two methods in that it accurately segmented the vascular regions and minimized the background noise.

Image segmentation with clustering algorithms was easily affected by the image background, resulting in a high level of noise. Consequently, subsequently applied algorithms could not correctly identify the vessel locations. Segmentation performance was better when the region was small. However, if vessels accounted for only a small part of a subimage, noise increased. Regarding the proposed flood fill algorithm, vascular subimages were first segmented. Next, with them as the center, threshold values were applied to the eight adjacent subimages for segmentation. This method effectively reduced noise, thereby facilitating subsequent algorithm implementation.

Using these three algorithms, 149 images were subjected to binary segmentation. The number of vascular regions (black pixels) and background regions (white pixels) was calculated. Table 5 presents the experimental results. Image segmentation of single subimages was conducted. The number of black pixels exceeded the corresponding number in the other two methods, demonstrating that background noise was not effectively removed. When the proposed flood fill algorithm was used, the number of black pixels was approximately 1.7 million fewer than that obtained through the first method. The comprehensiveness of the main blood vessels identified under the two methods was comparable. Overall, background noise was clearly better managed with the flood fill algorithm than with the single subimage processing method.

**Table 5:** Pixel number statistics of the three regional parameter expansion strategies

Region size/pixel number	Number of black pixels	Number of white pixels
Single sub-image image segmentation	8,801,450	30,258,006
Integration of the center sub-image with the eight adjacent sub-images before image segmentation	7,970,925	31,088,531
Segmentation of the eight adjacent sub-images by using the flood fill algorithm image segmentation	7,082,749	31,976,707

### Impacts of cover rules on vessel extraction

**Fig. 14:**
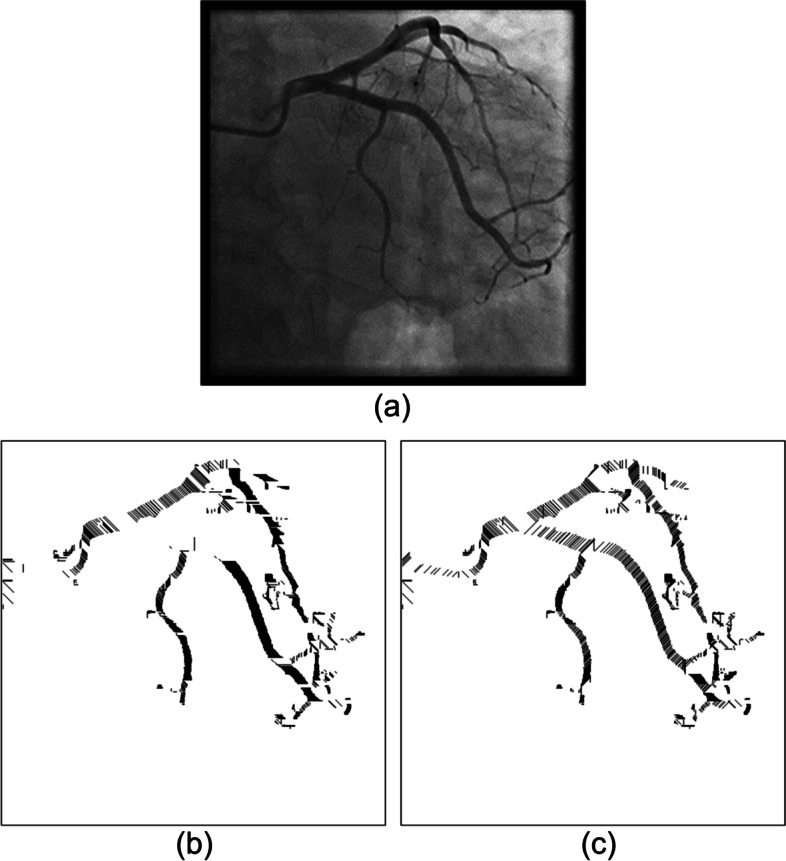
The impacts of cover type, (**a**) original images, (**b**) results from the use of three cover types. The LAD artery is not covered, so the diameter cannot be calculated. (**c**) Results from the use of five cover types

**Fig. 15:**
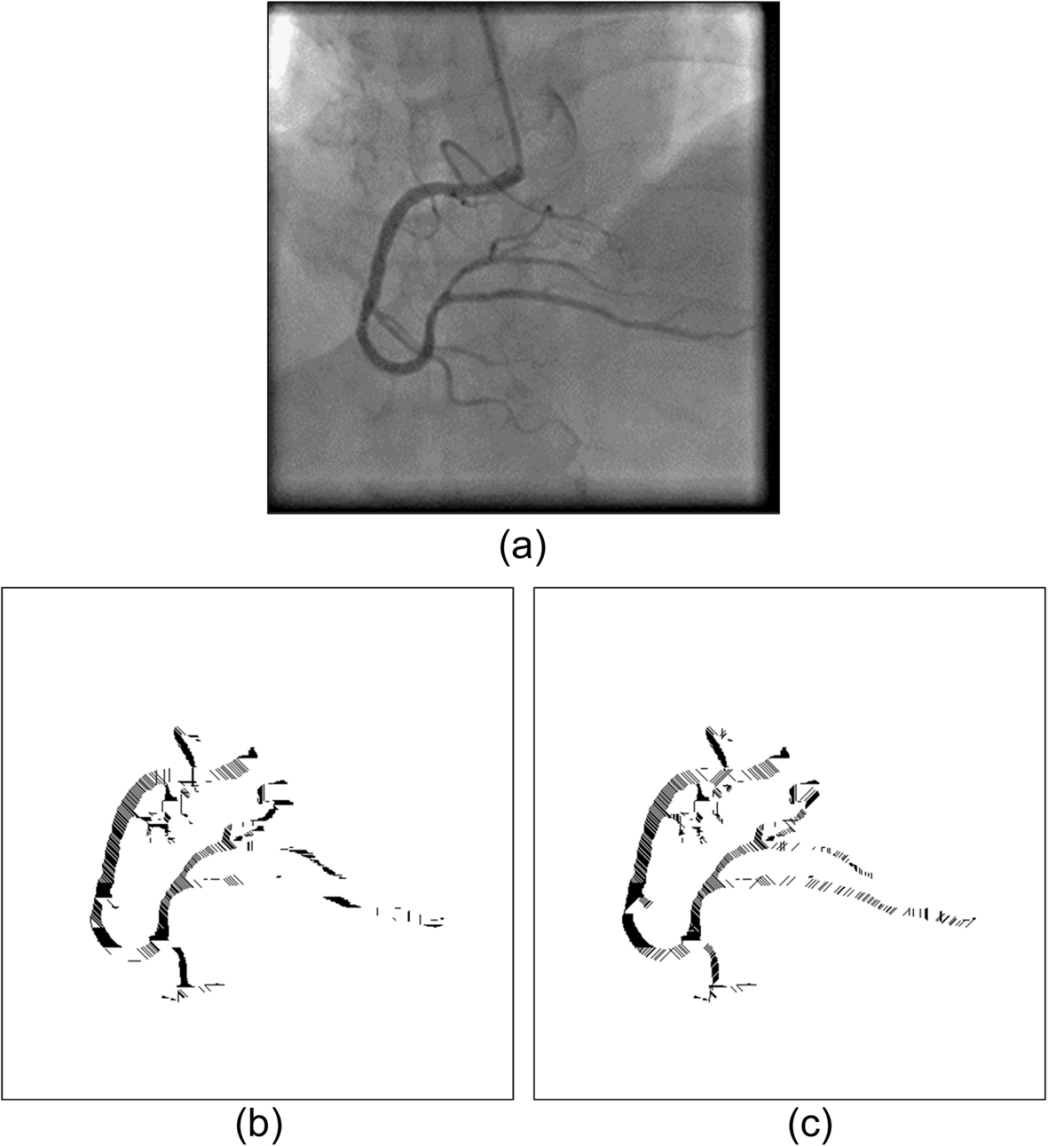
The impacts of cover type, (**a**) original images, (**b**) results from the use of three cover types. This method cannot correctly calculate the diameter of the blood vessel at the bottom. (**c**) Results from the use of five cover types

We examined the impacts of cover rules on the vessel extraction results. First, the impacts of cover type were analyzed. Two algorithms were compared. The first involved three cover types (horizontal, downward vertical, and bottom right diagonal). The second involved five cover types (horizontal, downward vertical, upward vertical, bottom right diagonal, and top right diagonal). Figures 14 and 15 present the comparison results, where (a) is the original image and (b) shows the experimental results from the use of three cover types. Regarding the location of the LAD artery in Fig. 14(b), because the blood vessels were not covered, their diameter could not be calculated. In (c), the use of five cover types resolved the problem of noncoverage of large vascular regions of the LAD artery. As shown in Fig. 15(b), at the bottom of the blood vessel where it curves, the bottom right diagonal cover was selected when the three cover algorithm was used. However, this was not the optimal cover method for vessel diameter calculation. As shown in part (c) of the figure, the use of the five-cover algorithm substantially mitigated the problem of noncoverage of the bottom part of the curved blood vessel, facilitating vessel diameter calculation.

Increasing the cover directions can resolve the problems with covering the bottom of curved blood vessels, with regard to both noncoverage and incorrect coverage. However, as shown in Fig. 14(b), a region was mistakenly covered at the crossing location of the left main coronary artery. This is because the two vessels overlapped when this image was captured. When calculating the optimal cover, the algorithm could not identify the overlapping region and used the top right cover. This overlapping problem can be resolved in the future by considering the analysis of images captured from different angles.

**Fig. 16:**
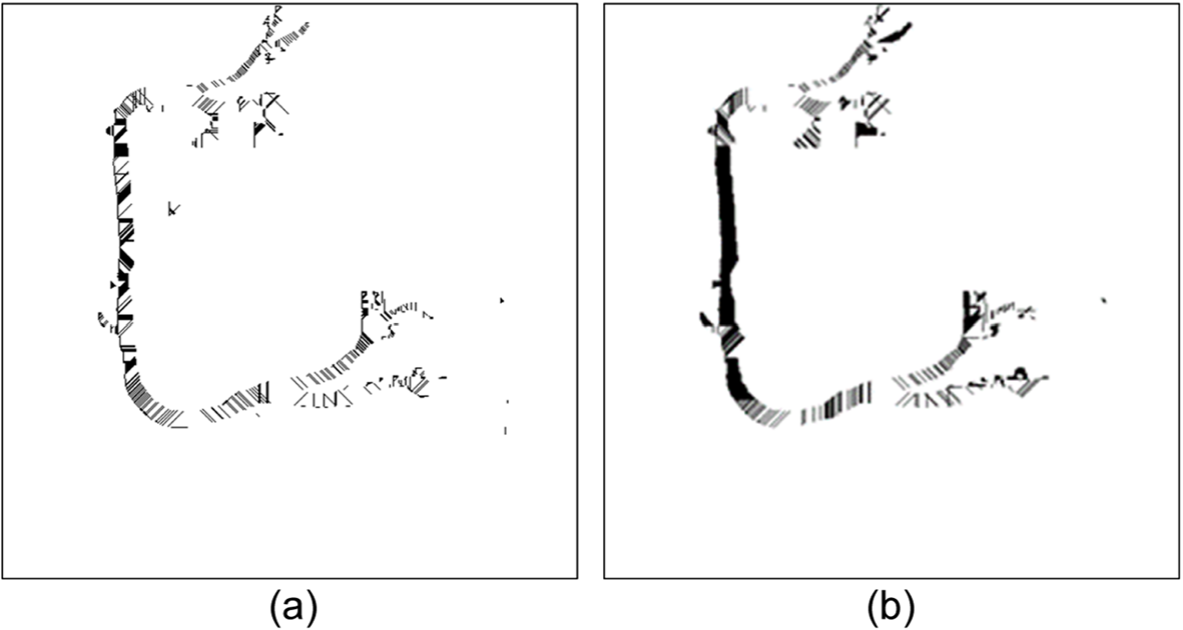
The impacts of the cover selected conditions in the OCT algorithm. (**a**) Only the shortest cover from the edge of each pixel was considered. The unevenness of the blood vessel edge will cause the coverage direction to be complicated, thereby affecting the accuracy of tracking the blood vessel diameter change. (**b**) Application results of the cover selection conditions

Binarized images often have rough, jagged edges caused by noise. This noise can lead to errors in the calculation of the shortest cover. For example, the main vessel observable in Fig. 16(a) is clearly vertical. However, its cover direction was complex, and the actual direction of the blood vessel was difficult to identify, which affected the data used for tracking the vessel diameter changes.

**Fig. 17:**
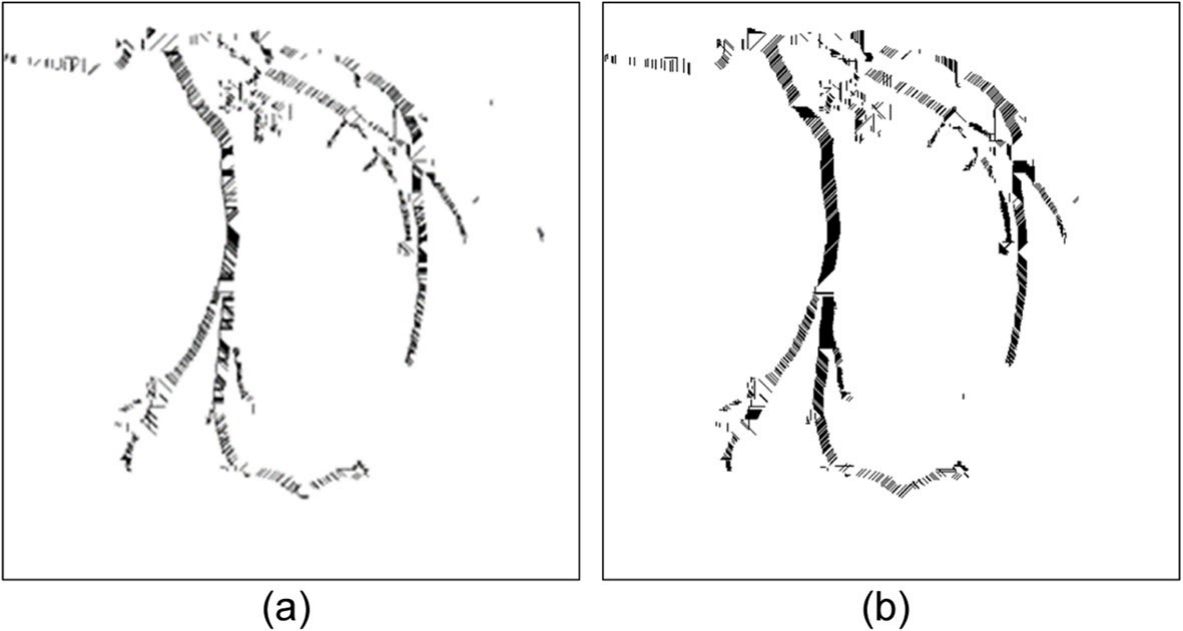
The impacts of the cover selected conditions in the OCT algorithm, (**a**) without using the rules, (**b**) application results of the cover selection conditions

To resolve this problem, we added rules (1) and (2) to the optimal cover algorithm and conducted experiments. The impacts of using and not using the rules were compared, as shown in Figs. 16-17. The left side of Fig. (a) presents the experimental results without the application of rules for covering angle and length differences, whereas the right side of Fig. (b) presents the results with these rules applied. After rule implementation, the cover density was higher, and the cover direction angles corresponded with the vessel diameter.

## Conclusion

Applying image segmentation techniques to medical image processing can facilitate the identification of lesions as well as diagnosis and treatment decision-making. However, they do not yield favorable results in application to coronary angiography involving catheterization. This is because factors (e.g., tissues) contribute to regional light and shadow changes during imaging that complicate the accurate extraction of vascular images in image segmentation. Regional parameter expansion effectively reduced background noise and clearly segmented the vessel paths. The small blood vessels were indicated by colors that were closer to the background and thus were not easily segmented. Overall, however, favorable experimental results were obtained.

Image segmentation can isolate vessel-containing image areas. To evaluate the severity of a lesion, its location and the changes in the diameter of the affected vessel must be known. As mentioned, the cover tree algorithm proposed in this study recorded these changes and information on the extension direction of the vessels through a tree structure. The algorithm calculated the shortest tangent of the blood vessel. Subsequently, the optimal cover was selected to cover the tangent. The cover length was then used to estimate the diameter of the blood vessel. Finally, spanning trees were used to record the adjacent covers, completing the construction of the vascular path tree. Variations in diameter length are indicated by different colors to facilitate the observation of vessel diameter changes at each segment. This method yielded favorable results with regard to measuring diameter changes in the major blood vessels. The optimal cover did not correspond to the tangent direction in some parts where the angle of the vessel curve was overly wide or smooth. Nonetheless, this method was simple and effective overall in both the theoretical and practical contexts. Future research directions can involve integrating the coronary artery disease grading system and quantitative indexes with the present algorithm for lesion classification. In practice, our method can be used to assess the type and severity of cardiac disease, including conditions affecting the coronary arteries.

## Data Availability

The data used to support the findings of this study are available from the corresponding author upon request.
